# Early Life Exposure to Aflatoxin B1 in Rats: Alterations in Lipids, Hormones, and DNA Methylation among the Offspring

**DOI:** 10.3390/ijerph18020589

**Published:** 2021-01-12

**Authors:** Oluwakemi A. Rotimi, Chinonye D. Onuzulu, Alisa L. Dewald, Jessa Ehlinger, Isaacson B. Adelani, Olutola E. Olasehinde, Solomon O. Rotimi, Jaclyn M. Goodrich

**Affiliations:** 1 Department of Biochemistry, Covenant University, Ota 112233, Ogun State, Nigeria; onuzuluc@myumanitoba.ca (C.D.O.); bababode.adelani@covenantuniversity.edu.ng (I.B.A.); olutola.olasehinde@stu.cu.edu.ng (O.E.O.); ola.rotimi@covenantuniversity.edu.ng (S.O.R.); 2 Department of Biochemistry and Medical Genetics, University of Manitoba, Winnipeg, MB R3T 2N2, Canada; 3 Department of Environmental Health Sciences, University of Michigan School of Public Health, Ann Arbor, MI 48109, USA; aldewald@umich.edu (A.L.D.); ejessa@umich.edu (J.E.); gaydojac@umich.edu (J.M.G.)

**Keywords:** aflatoxin B1, liver, lipid, hormones, DNA methylation, developmental origins of health and disease, gestational exposure

## Abstract

Aflatoxins are toxic compounds produced by molds of the *Aspergillus* species that contaminate food primarily in tropical countries. The most toxic aflatoxin, aflatoxin B1 (AFB1), is a major cause of hepatocellular carcinoma (HCC) in these countries. In sub-Saharan Africa, aflatoxin contamination is common, and perinatal AFB1 exposure has been linked to the early onset of HCC. Epigenetic programming, including changes to DNA methylation, is one mechanism by which early life exposures can lead to adult disease. This study aims to elucidate whether perinatal AFB1 exposure alters markers of offspring health including weight, lipid, and hormone profiles as well as epigenetic regulation that may later influence cancer risk. Pregnant rats were exposed to two doses of AFB1 (low 0.5 and high 5 mg/kg) before conception, throughout pregnancy, and while weaning and compared to an unexposed group. Offspring from each group were followed to 3 weeks or 3 months of age, and their blood and liver samples were collected. Body weights and lipids were assessed at 3 weeks and 3 months while reproductive, gonadotropic, and thyroid hormones were assessed at 3 months. Prenatal AFB1 (high dose) exposure resulted in significant 16.3%, 31.6%, and 7.5% decreases in weight of the offspring at birth, 3 weeks, and 3 months, respectively. Both doses of exposure altered lipid and hormone profiles. Pyrosequencing was used to quantify percent DNA methylation at tumor suppressor gene *Tp53* and growth-regulator *H19* in DNA from liver and blood. Results were compared between the control and AFB1 exposure groups in 3-week liver samples and 3-week and 3-month blood samples. Relative to controls, *Tp53* DNA methylation in both low- and high-dose exposed rats was significantly decreased in liver samples and increased in the blood (*p* < 0.05 in linear mixed models). *H19* methylation was higher in the liver from low- and high-exposed rats and decreased in 3-month blood samples from the high exposure group (*p* < 0.05). Further research is warranted to determine whether such hormone, lipid, and epigenetic alterations from AFB1 exposure early in life play a role in the development of early-onset HCC.

## 1. Introduction

The developmental origins of health and disease (DOHaD) paradigm posits that environmental conditions in gestation and early postnatal life set the stage for the health trajectory of offspring throughout their lifetime [1]. Early evidence for DOHaD included studies showing that adults were at a much higher risk for coronary heart disease or ischemic heart disease if they were small at birth and remained small in the first two years of life [1,2]; rapid weight gain and catch-up growth later in childhood seemed to underlie some of this risk [2]. Decades of research in this field to date have shown that environmental factors ranging from macro-and micronutrient availability to chemical and biological agent exposures early in life can lead to fetal and perinatal adaptive responses that impact development, growth, metabolism, and ultimately ‘program’ the offspring for adult disease. In addition to coronary and other metabolic diseases, evidence now shows that the early life environment can also increase the risk for the development of cancer, especially hormone-dependent cancers [3]. Birthweight is often a proxy for the impact of the gestational environment and a predictor for health in years to come; a recent systematic review suggests that high birth weight is associated with overall and prostate cancer mortality [4]. Low birth weight has been shown to be associated with hepatoblastoma [5,6], liver diseases, and HCC in adulthood [7,8].

One of the many biological mechanisms that are at play in the DOHaD is epigenetic alteration. Here we define epigenetic modifications as mitotically and in some cases, meiotically heritable modifications to DNA and chromatin that control the expression of genes without altering the DNA sequence [9,10]. Aberrant epigenetic gene regulation has been proposed as a mechanism of action for non-genotoxic carcinogenesis [11] and is also important for fetal and childhood growth and development, including the imprinted genes *Igf2* and *H19* and non-imprinted gene *Igf1* [12,13,14]. The epigenome is vulnerable to deregulation during embryogenesis due to the waves of epigenetic erasure (i.e., demethylation post-fertilization) and post-implantation reprogramming that occur [10]. Disruption during this reprogramming stage can be passed on to all subsequent cells across tissue types. DNA methylation is one mechanism of epigenetic regulation that is stable across time at many cytosine-guanine (CpG) dinucleotides [15]. Environmental exposures, especially during early gestation, may perturb epigenetic programming and set the stage for disease development later in life; exposures to chemicals such as bisphenols and phthalates have been shown to alter the offspring epigenome [16,17,18]. Recent studies have implicated naturally-occurring mycotoxins, including aflatoxin B1 (AFB1) as epigenetic-modifying exposures [19,20,21,22,23].

Aflatoxins are secondary metabolite products of *Aspergillus* species molds, and human exposure to these toxins occurs due to exposure to contaminated food such as grains, nuts, eggs, spices, milk, and meat in tropical countries of sub-Saharan Africa and southeast Asia [24,25]. Although aflatoxin contamination is common and a major problem in these countries, studies have shown that aflatoxin contamination is a global occurrence including in parts of Europe and the Americas [26,27,28]. AFB1 is the most toxic and common of all types of aflatoxins in human populations. AFB1-exo-8,9-epoxide, the highly reactive metabolite of AFB1 which binds to DNA and forms adducts, has been linked to hepatotoxicity and carcinogenesis [29,30,31]. Liver cancer is the fourth most frequent cancer in sub-Saharan Africa [32], and the increase of liver cancer in this region has been associated with exposures to both hepatitis B virus and AFB1 [33]. In addition to DNA adduct formation, there is evidence for an epigenetic change in the liver following exposure to AFB1 among adults [20,21]. African children are known to be exposed to AFB1 during gestation, breastfeeding, and through grain-based weaning foods [34]. While early-life exposure has been linked to growth faltering [34] and changes to DNA methylation in one epidemiological study [19], this is still a new area of research that has not been studied in animal or human models of chronic exposure with long-term follow-up past birth. 

The early onset of carcinogenesis involves epigenetic changes in tumor suppressor genes [20,35]. *Tp53* encodes p53, a tumor suppressor known for its role in cancer prevention [36,37]. Apart from this role, studies now show that it plays additional functions, including regulation of liver metabolism and homeostasis [38]. P53 is also involved in lipid and hormone metabolic pathways [38,39,40]. There is evidence suggesting interactions between p53 and hormones could be involved in carcinogenesis [38,40,41,42]. Previous studies from our lab and others have shown that AFB1 dysregulates the metabolism of lipids [43,44,45] and hormones [46,47,48] in rats. Growing evidence implicates imprinted genes, including *Igf2* and *H19,* which are typically known for their roles in early-life growth, to contribute to risk for cancers [49,50,51].

In this study, we seek to understand the effect of two doses of AFB1 exposure using a perinatal rat exposure model (gestation through weaning) on epigenetic programming in the offspring and other physiological indicators of health and disease risk (hormone levels, lipid levels, and weight). Specifically, we assess DNA methylation at *Tp53*, a crucial tumor suppressor gene, and *H19*, an environmentally-responsive imprinted gene that has been linked to HCC risk. DNA methylation at these genes was quantified immediately following cessation of exposure (3 weeks of age) and in early adulthood (3 months of age) in the liver, the target tissue of interest, as well as blood, the surrogate tissue most often accessible in human studies. We also measured weight, cholesterol, triglycerides, and hormones. We hypothesized that perinatal exposure to AFB1 would reduce offspring growth, decrease hormone and lipid levels, and alter DNA methylation. We also hypothesized that these changes would be detected in both liver and blood and persist until adulthood after exposure ceases.

## 2. Materials and Methods

### 2.1. Animals

Ethical approval for the use of animals in this research was given by Covenant University Health Research Ethics Committee (protocol number CHREC/026/2018), and all procedures were in strict compliance with set ethical standards. Eighteen 6-week-old inbred female and nine male Wistar rats weighing between 100 and 120g were purchased from Lagos University Teaching Hospital (LUTH), Lagos, Nigeria, and used for this experiment. Animals were housed in clean, well-ventilated cages for standard 12-h light and dark cycles at room temperature in the animal house in Covenant University, Nigeria. Animals were fed standard rat chow and clean water *ad libitum* and allowed to acclimatize for 3 weeks prior to the start of the experiment.

### 2.2. Chemicals

AFB1 was obtained from Sigma-Aldrich (St. Louis, MI, USA) while kits for hormone and lipid profiles were products of Biobase (Jinan, China) and Randox (Crumlin, UK) respectively.

### 2.3. Experimental Design

Female animals were randomly distributed into 3 groups of 6 animals each and administered feed containing one of two doses (0.5 or 5.0 mg/kg feed) of AFB1 while the third group served as control. The doses were chosen based on the concentrations of AFB1 found in maize grains in Nigeria [52]. Mating was achieved by placing one male animal in a cage with two females and ended once a vaginal plug was detected. AFB1 treatment began two weeks prior to mating and lasted throughout pregnancy, ending precisely three weeks after the birth of the offspring. This is a perinatal exposure model that has been employed in other environmental epigenetic research studies [16]. To ensure that animals maintained a healthy weight, body weights were measured biweekly throughout the experiment, up until the time of euthanasia. At weaning (3 weeks), half of the litters were weighed and anesthetized using 50 mg ketamine/5 mg xylazine followed by euthanasia using a protocol approved by the Covenant University Ethics Committee (protocol number CHREC/026/2018). Blood was collected via cardiac puncture using syringes coated with lithium heparin. Whole blood was centrifuged at 3000 rpm for 15 minutes to obtain plasma which was used for hormonal assays and lipid profile analysis. The whole liver was excised, rinsed in phosphate buffer saline, and frozen for further analysis. The remaining offspring were fed a diet free of AFB1 up to 3 months of age, at which point they were also euthanized for blood and liver collection.

### 2.4. Hormone and Lipid Analysis

The hormone profile (testosterone, progesterone, estradiol, follicle-stimulating hormone (FSH), luteinizing hormone (LH), prolactin, thyroxine (T4), and triiodothyronine (T3)) was assessed in the plasma using ELISA kits purchased from Biobase (Jinan, China), and following the manufacturer’s instructions. Briefly, samples or standards were added to microplate wells previously coated with specific antibody after which horseradish peroxidase conjugate was added and incubated at 37 °C for one hour. After incubation, wells were washed five times and chromogen was added for color formation. Absorbance was measured spectrophotometrically at 450 nm using a plate reader. Total plasma cholesterol and triglycerides were measured using commercially available kits (Randox).

### 2.5. DNA Methylation Analysis

High-molecular-weight DNA was extracted from 3-week and 3-month liver and blood samples using DNA DNeasy^®^ Blood and Tissue extraction (Qiagen, Germantown, MD, USA) kits according to the protocol of the manufacturer. DNA samples were shipped on dry ice to the University of Michigan and stored at −80 °C until analysis. DNA samples were then treated with sodium bisulfite using kits from Zymo Research (Irvine, CA, USA) [53]. DNA methylation was quantitatively analyzed at cytosine-phosphate-guanine dinucleotides (CpG sites) via pyrosequencing [54] at *Tp53* (9 CpG sites) and *H19* (6 sites). Primer designs were modified using PyroMark Assay Design Software 2.0 (Qiagen, Germantown, MD, USA) to amplify regions in the promoters of *Tp53* and *H19* based on assays previously used by others (see primer sequences in Supplemental Table S1) [13,55]. The *H19* assay covers the second imprinting control region (ICR2) of the gene. For all genes, sequences were amplified with HotStarTaq Master Mix (Qiagen) from approximately 50 ng bisulfite-converted DNA. At least four replicates of commercial rat DNA and negative controls were included in each batch (96-well PCR/pyrosequencing plate). The percentage of methylated cells was quantified by a PyroMark ID Pyrosequencer (Qiagen). Pyro Q-CpG Software computes percent methylation and performs internal quality control checks to ensure complete bisulfite conversion, adequate signal over background noise, etc. Only samples and CpG sites passing quality control were used for downstream statistical analyses. A subset of samples (>10%) were run in duplicate, and duplicate reads were averaged when available.

### 2.6. Statistical Analysis

Descriptive statistics were calculated first for all continuous variables (weights, site-specific DNA methylation), and frequencies for categorical variables. Two-sided t-tests assuming unequal variances were conducted to compare birth weight and weight at sacrifice between all offspring and sex-stratified offspring for the following comparisons: control vs. low exposure group, control vs. high, and low vs. high. Mixed-effects regression was also performed on weight as a repeated measure (stages: at birth, 3 weeks, and 3 months), adjusting for sex. An interaction term between stage and exposure group was tested as well as a random intercept for a dam. The latter did not improve the model fit and was excluded in the final model. Hormone and lipid data were analyzed using SPSS v23 (IBM Corp., Armonk, NY, USA). One-way analysis of variance (ANOVA) with post-hoc tests was used to compare the means in each exposure group.

Before analysis of DNA methylation data, CpG sites and/or samples that failed quality control checks were removed. DNA quality from one set of samples (3-month liver) was low, and most of these samples failed quality control. Thus, 3-month livers were excluded from statistical analyses. For *Tp53*, DNA methylation data had a higher passing rate for the first 6 CpG sites in the assay. As such, only samples that passed for all 6 CpG sites were included in further analyses. These 6 CpG sites were highly correlated with one another (Spearman rho > 0.5 for intra-site comparisons with most >0.75), and we averaged them together. The six CpG sites of *H19* were highly correlated (Spearman rho > 0.7), and we averaged the sites together for most statistical analyses. We first compared average values for each gene across all exposure groups within 3-week livers, 3-week blood, and 3-month blood samples separately using ANOVA. We utilized Welch’s F-test instead if variances between groups were not homogenous according to Levene’s test. To better capture intra-gene variability at each CpG site, we performed mixed-effects regression, [56] where the outcome is a repeated measure of DNA methylation at multiple CpG sites. Based on the correlation structure between the sites, we assumed an autoregressive covariance structure between *Tp53* sites (all 9 CpG sites included) and compound symmetry for *H19* (all 6 CpG sites). Models were run separately for liver (adjusting for CpG site and sex) and blood (adjusting for CpG site, sample time point, and sex) data with exposure group as the predictor of interest. We also included a random intercept for litter in *H19* methylation models as litter explained a portion of the covariance for *H19* but not for *Tp53*.

Unless otherwise specified, statistical analyses were conducted in SAS version 9.4 (SAS Institute Inc., Cary, NC, USA), and *p*-values < 0.05 were considered statistically significant.

## 3. Results

### 3.1. Weight Gain/Weight Status of the Rodent Population

Offspring from the unexposed control group (41 from 6 dams), the low dose exposure group (48 from 5 dams), and the high dose exposure group (36 from 4 dams) were followed up to either 3 weeks of age or 3 months of age. Weights were measured at birth and at the time of sacrifice. At birth, offspring of the high exposure group were significantly lower in weight by 0.8g and 1.3g for females and males, respectively (Table 1) compared to the control group. At 3 weeks of age, offspring from both the low and high exposure groups were statistically significantly lower in weight than the control group when comparing all offspring or males and females separately. At 3 months of age, the control group still had the highest weights. Statistically significant differences were noted when comparing the low group to the control group or the low to the high group. When modeling all weight data from 125 offspring as a repeated measure, adjusting for sex and stage, the exposure group was still statistically significant (*p* < 0.0001 for fixed effect of exposure and for interaction between exposure-group and timing).

### 3.2. Lipids

Total cholesterol and triglycerides were measured in plasma samples from 3 weeks and 3 months animals. Within each exposure group, cholesterol and triglycerides decreased as the animals aged (Table 2). At the time exposure ceased (3 weeks), cholesterol levels were 32% and 36% lower in the low and high exposure groups, respectively, compared to controls (*p* < 0.05). At 3 months of age, differences remained though, at that time, both exposure groups had 19% lower cholesterol compared with controls (*p* < 0.05). At 3 weeks, triglycerides were significantly higher in low and high exposure groups (14% and 18%, respectively, *p* < 0.05). At 3 months of age, the low exposure group was no longer significantly different from the control group, yet the high exposure group retained a 19% higher level of triglycerides compared to controls. However, when we stratified by sex, the higher exposure group had significantly higher triglycerides in both males and females while the low exposure group of male rats had a significantly lower triglyceride level compared with control at both time points (stratified results not shown).

### 3.3. Hormonal Changes at 3 Months of Age

**Figure 1:**
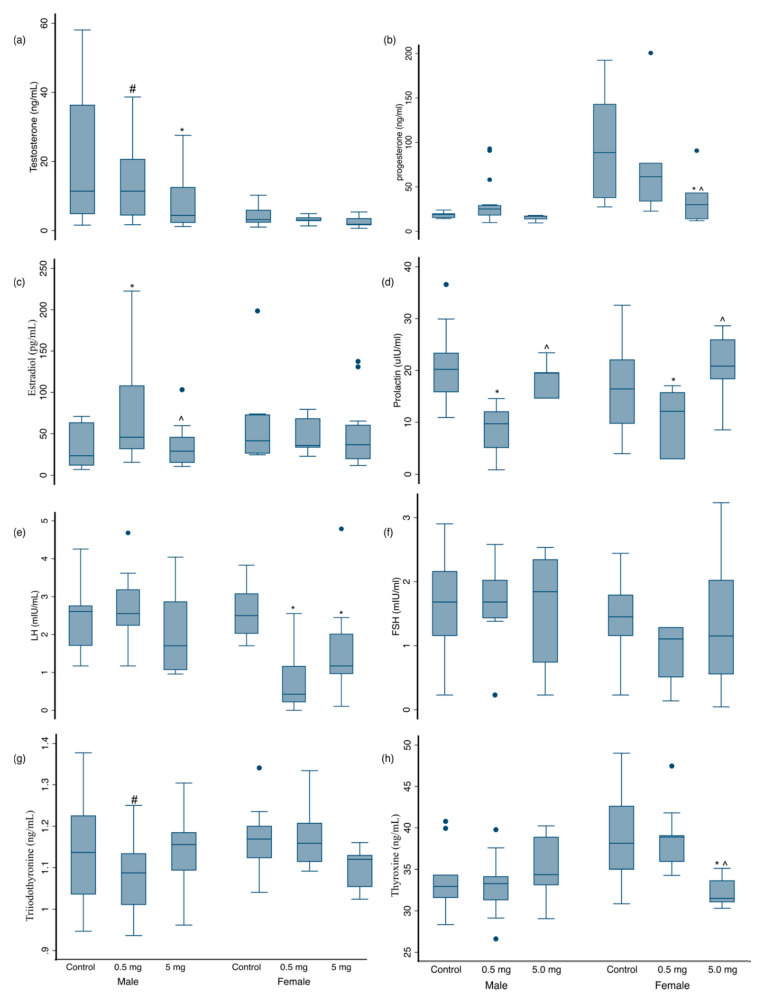
Early Life AFB1 Exposure and Hormones in 3-month Offspring. Distributions of plasma hormones at 3 months of age are shown for male and female offspring of the unexposed (control) and low- and high-exposure groups for the following hormones: (**a**) Testosterone, (**b**) Progesterone, (**c**) Estradiol, (**d**) Prolactin, (**e**) Luteinizing hormone (LH), (**f**) Follicle-stimulating hormone (FSH), (**g**) Triiodothyronine (T3), and (**h**) Thyroxine (T4). Boxes represent the interquartile range (IQR) while the line inside each box represents the median. The whiskers represent 1.5 × IQR while the dots are outliers. * Denotes significantly different from control at *p* < 0.05, ^#^ significantly different from control at *p* < 0.01, ^ significantly different from the low exposure group at *p* < 0.05.

We observed statistically significant differences between exposure groups when comparing reproductive, gonadotropic, and thyroid hormone (Figure 1) levels in 3-month plasma samples, stratified by sex. Among males, testosterone was lower in the low (*p* < 0.1) and high (*p* < 0.05) exposure groups compared with the control group. Estradiol was higher and prolactin lower in low exposure males compared to both control and high exposure groups (*p* < 0.05). Among females, LH was lower in both exposure groups compared to controls (*p* < 0.05). Progesterone was lower in high exposure females only (*p* < 0.05) and prolactin was decreased in low exposure group males and females compared to controls. When assessing thyroid hormones, T4 was decreased in the high exposure group females compared to controls and the low dose group (*p* < 0.05). T3 was decreased in males in the low exposure group (*p* < 0.01).

### 3.4. DNA Methylation in Liver and Blood

We observed exposure-dependent differences in *Tp53* and *H19* DNA methylation in the liver at 3-weeks of age (Table 3) and in blood at 3-weeks and 3-months (Table 4). In the liver, the target tissue of interest, *Tp53* methylation was lower in both exposure groups compared to the unexposed group (ANOVA *p*-value = 0.06). In repeated measures linear regression of 9 CpG sites of *Tp53*, taking into account their covariance and adjusting for sex and CpG site, *Tp53* methylation was statistically significantly lower in both exposure groups compared to unexposed (estimate of −1.09 ± 0.34%, *p* = 0.002 for low and −0.91 ± 0.34%, *p* = 0.01 for high). *H19* methylation was higher in low and high exposure groups’ liver samples compared to controls (ANOVA *p* = 0.0041). In repeated measures linear regression of 6 CpG sites adjusting for sex, site, and dam, these differences remained statistically significant for the low (effect estimate 5.37 ± 2.56%, *p* = 0.04) and high (6.88 ± 2.64%, *p* = 0.0098) exposure groups.

In blood, *Tp53* methylation was higher in exposed versus control rats at both 3 weeks and 3 months, yet this was only statistically significant at 3 months of age (ANOVA *p* = 0.07 for 3 weeks and *p* < 0.001 for 3 months). In repeated measures regression, this difference was statistically significant in both exposure groups and at both time points (see Table 4). There was some evidence for reduced blood DNA methylation at *H19*, though this was only statistically significant at 3 months of age (ANOVA *p* = 0.04) with effect estimates in the adjusted regression model of −10.7 ± 6.05%, *p* = 0.08 for low and −12.8 ± 6.59%, *p* = 0.05 for high exposure groups compared to controls.

## 4. Discussion

In a rat study of perinatal exposure to two doses of AFB1, we observed effects on the offspring with both doses at weaning (3 weeks of age) and also into adulthood (3 months) after exposure had ceased. Perinatal AFB1 exposure was associated with decreased weight (at birth, 3 weeks, and 3 months), altered lipids (decreased cholesterol at 3 weeks and 3 months, increased triglycerides at 3 weeks), and altered hormones (decreased testosterone, progesterone, and LH) at *p* < 0.05. Both doses of perinatal AFB1 exposure were associated with decreased methylation at the *Tp53* promoter and increased methylation at the *H19* ICR in 3-week liver samples. In blood samples, associations (*p* < 0.05) were observed between exposure and increased *Tp53* methylation (3-weeks and 3-months) and decreased *H19* methylation (3-months).

Our results showed that prenatal exposure to AFB1 decreased the weight of the animals at birth and weight gain until 3 months of age. These results indicate that prenatal AFB1 exposure can decrease birth weight and affect the weight gain of the animals, even after cessation of the exposure. The results of this study are in agreement with other studies [57,58,59]. Supriya et al. found a dose-dependent decrease in birth weight of rats prenatally exposed to AFB1 during gestational days 12–19 of pregnancy [58,59]. A human study conducted in The Gambia found inverse linear associations between maternal AFB1 concentrations and weight and height of the offspring in the first year of life [60]. Some studies have also shown that low birth weight may increase the risk of liver diseases and HCC in adulthood. For example, a large cohort study of Danish children born between 1936–1980 [7] reported a positive association between the risk estimates of low birth weight and primary liver cancer and HCC in men. In women, the risk was associated with both low and high birth weight. Similar results were also observed in a Finnish birth cohort; Sandboge et al. [8] concluded that small birth size increased the odds of adult non-alcoholic fatty liver disease.

To the best of our knowledge, this is the first study to examine the effects of prenatal AFB1 exposure on cholesterol and triglyceride concentrations in rats at weaning (3 weeks) and after cessation of exposure in adulthood (3 months). Our results showed that prenatal AFB1 exposure increased triglyceride concentrations at 3 weeks (low and high exposure groups) and at 3 months (high exposure group). Interestingly, the high triglyceride concentrations observed were not correlated with body weight. Even so, the hypertriglyceridemia observed may have other implications for conditions including coronary heart disease, insulin resistance, or metabolic syndrome [61,62,63]. On the other hand, prenatal AFB1 exposure to both doses decreased cholesterol levels at 3 weeks and 3 months. These results differ from the response of cholesterol to AFB1 exposure in adulthood. Results from our laboratory and others show that AFB1 exposure in adult rats increases cholesterol levels [43,44,64,65]. This indicates that the mechanism of AFB1 toxicity in utero may differ from that of adulthood and therefore requires further investigation.

Cholesterol is important for the biosynthesis of steroid hormones [48,66]. In this study, prenatal AFB1 exposure significantly decreased concentrations of testosterone and progesterone in male and female rats, respectively, and this could have resulted from reduced cholesterol levels. Apart from the reduced cholesterol levels, the response of steroid hormones to prenatal AFB1 exposure in this study was sex-dependent, which may result from the influence of AFB1 on sex and developmental-stage specific differences in the synthesis of these hormones [67]. Additionally, sex differences have been observed in the expression of genes involved in steroid hormone metabolism and response [68]. The decrease in testosterone observed in our study agrees with other studies [46,59]. The study by Supriya and Reddy showed that prenatal AFB1 exposure resulted in decreased circulating testosterone levels in male rats [59]. This decrease could have resulted from reduced androgen biosynthesis as shown in their study by the reduction in the activities of 3ß-hydroxysteroid dehydrogenase and 17ß-hydroxysteroid dehydrogenase in rat testis. The reduction in testosterone may also be due to the ability of AFB1 to bind to Steroidogenic acute regulatory (StAR) protein which aids the transport of cholesterol from the outer to the inner mitochondrial membrane where it is converted to pregnenolone for testosterone synthesis [46]. The decreased levels of progesterone observed in female rats may be linked to the decreased level of LH since biosynthesis of steroid hormones is controlled by gonadotropic hormones such as LH and FSH. Specifically, LH and FSH are involved in the synthesis and regulation of steroid hormones: testosterone, progesterone, and estradiol [66].

In our study, LH levels were decreased in female rats, while FSH was not significantly different in male or female rats. Our results are different from that of Supriya and Reddy, where prenatal AFB1 exposure significantly increased LH and FSH. The differences between studies could be related to exposure timing (acute vs. chronic). While our exposure spanned from two weeks before mating, throughout pregnancy until weaning, theirs was limited to gestational days 12–19. AFB1 has also been shown to decrease LH in adult rats [69,70] and lactating buffalos [71]. Apart from the steroid and gonadotropic hormones, prenatal AFB1 exposure also decreased prolactin at the low exposure dose and thyroxine at the high exposure dose. Overall, the altered hormonal status of the exposed rats indicates that AFB1 could be an endocrine disruptor. Like other endocrine disruptors, its exposure in utero may increase the risk for non-communicable diseases later-in-life [72].

The results of this and other studies suggest that AFB1 is not only a genotoxic agent but also an epigenetic modifier. Widespread epigenetic aberrations including global hypomethylation and hypermethylation of tumor suppressor genes are one of the hallmarks of cancer [73], and some researchers even posit that epigenetic change contributes to all hallmarks of cancer [74]. Most studies to date on AFB1 and the epigenome have used clinical tumor samples or in vitro models (i.e., human hepatocytes). The in vitro studies observed changes to DNA methylation, miRNA expression, and gene expression following AFB1 exposure in human primary hepatocytes or hepatic stem cells (HepaRG) [33,75,76,77]. In tumor samples from HCC patients, DNA methylation levels at repetitive elements and genes including *GSTP1*, *RASSF1A*, and *p16* have been correlated with biomarkers of AFB1 exposure [20,21,78,79]. There is growing evidence that gestational exposures can alter epigenetic programming and thereby increase the risk for the development of cancers later in life. Most of the evidence to date centers around endocrine-disrupting chemicals and the development of reproductive tract, breast, and prostate cancers [3].

To our knowledge, ours is the first study to assess whether early life AFB1 exposure modifies the epigenome into adulthood, potentially contributing to risk for HCC or other cancers. We observed differences by low and high AFB1 exposure in liver and blood at the tumor suppressor gene, *Tp53*, and at the imprinted gene, *H19*. One epidemiological study of mother-infant pairs from The Gambia reported associations between early gestation AFB1 and DNA methylation of infant samples using an epigenome-wide approach at 71 CpG sites including genes involved in growth and immune function [19]. In this study, we selected two genes for analysis that are relevant to carcinogenesis and have evidence for environmental liability by early-life exposures. *Tp53* is a well-characterized tumor suppressor gene, and mutations in this gene have been linked to HCC along with other cancers [80,81]. The promoter region of *Tp53* assessed in this study was previously shown to be responsive to a gestational diet of malnutrition. In offspring with gestational exposure to reduced protein, *Tp53* methylation was reduced, and protein expression increased in the kidney at 2 and 3 months of age. Interestingly, this was reversed when folic acid supplementation was given after weaning [55], highlighting the dynamic nature of epigenetics and the possibility for interventions to improve health trajectories. *H19* is an imprinted gene important to regulating fetal growth; DNA methylation at the regulatory ICRs of this gene is modifiable by prenatal exposures to environmental chemicals and diet [82,83,84,85]. In healthy tissue, *H19* expression is downregulated after birth, but it reappears in many tumors. *H19* is now widely believed to be an oncogene that contributes to tumor initiation and progression for many cancers [50,51] including HCC [86]. This study provides evidence for perinatal AFB1 exposure and altered liver and blood DNA methylation of *H19* in rats. Whether this early life epigenetic programming influences downstream cancer risk in rats, and more importantly in humans, is yet unknown.

In this study, aflatoxin exposure led to decreased *Tp53* methylation in the liver, yet increased in the blood. Likewise, the direction of effect on *H19* methylation from AFB1 exposure was opposite for liver and blood. In human studies, when target tissues are not accessible, blood DNA methylation is often used to approximate effects in tissues of interest. In this study, both tissues did have significant DNA methylation changes by exposure. However, given the difference in the direction of effect, using blood to predict effects in the liver is not appropriate for every gene. Results in blood-only studies need to be interpreted with caution.

This study had several advantages, including the modeling of a longer-term and daily chronic AFB1 exposure from pre-conception through lactation and follow-up of the offspring into adulthood. In addition to weight, we assessed hormone levels, lipids, and epigenetics in two tissues. The use of blood (a surrogate tissue) in addition to the target tissue (liver) of interest is relevant because human epigenetic studies are typically restricted to the use of easily accessible surrogate tissues such as saliva or blood. This study had limitations, including a small sample size within each exposure group and the inability to perform epigenetic analysis on the 3-month liver samples. Functional validation was not performed to assess whether epigenetic changes led to changes in gene or protein expression. We only assessed two genes, selected by a hypothesis-driven approach; many more genes could be dysregulated by AFB1 exposure that are relevant to developmental processes and cancer risk. While the 3-month follow-up was a strength, we did not follow rodents into mid- or late-adulthood and assess the incidence of cancer.

## 5. Conclusions

In this study of daily AFB1 exposure from pre-conception through lactation, we report statistically significant effects in the offspring at 3 weeks and 3 months of age. Results from this study showed that prenatal exposure to AFB1 reduced body weight and disrupted lipid and hormone levels even after cessation of exposure. These changes could have implications for increasing the risk of diseases later in life. The mechanisms associated with these changes require further exploration. DNA methylation at two genes important for cancer risk and development was different in exposed rodents’ liver and blood samples. The direction of association between exposure and DNA methylation was opposite in liver and blood for both *H19* and *Tp53*, cautioning the interpretation of epidemiological studies that only have access to blood. Future research in rodent models of AFB1 exposure or human populations should perform epigenome-wide analysis coupled with transcriptomics and ideally a long-term follow-up to identify reprogrammed genes that contribute to HCC and other cancers. This knowledge will be critical to informing policymaking and risk assessment of the impacts of AFB1 through food contamination in vulnerable pregnant women and children.

## Figures and Tables

**Table 1:** Prenatal Aflatoxin B1 Exposure and Offspring Weight (g).

Exposure Group	Offspring	At Birth		3 Weeks		3 Months	
		Mean (SD)	N	Mean (SD)	N	Mean (SD)	N
Control	All	6.3 (1.5)	41	47.9 (5.1)	19	191 (28.8)	22
	Females	6.2 (1.5)	22	46.1 (3.6)	11	167 (13.7)	11
	Males	6.5 (1.6)	19	50.0 (6.0)	8	217 (12.7)	11
0.5 mg/kg	All	6.3 (1.1)	48	34.6 (6.7) **	19	155 (12.8) **	29
	Females	6.4 (1.2)	20	34.9 (7.4) *	9	158 (11.9)	11
	Males	6.3 (1.0)	28	34.3 (6.5) **	10	153 (13.4) **	18
5.0 mg/kg	All	5.3 (0.7) ** ^##^	36	32.7 (4.2) **	17	177 (33.6) ^#^	19
	Females	5.4 (0.8) * ^#^	21	33.8 (4.5) **	10	153 (15.8) *	11
	Males	5.2 (0.7) * ^##^	15	31.1 (3.4) **	7	209 (23.2) ^##^	8

* *p* < 0.05 and ** *p* < 0.001 when compared to control within same time period and offspring group (all, females or males). ^#^ *p* < 0.05 and ^##^ *p* < 0.001 when compared to low group within same time period and offspring group (all, females, or males).

**Table 2:** Cholesterol and Triglyceride Concentrations in Liver at 3 Weeks and 3 Months of Age by Exposure Group.

AFB1 Dose Group	Cholesterol (mg/dL)		Triglycerides (mg/dL)	
	3 Weeks	3 Months	3 Weeks	3 Months
Control (no AFB1)	299.16 ± 19.73 ^a^	184.60 ± 11.98 ^a#^	373.44 ± 36.72 ^a^	212.55 ± 12.39 ^a#^
0.5 mg/kg	204.91 ± 17.45 ^b^	148.85 ± 9.83 ^b#^	425.93 ± 25.44 ^b^	200.75 ± 12.50 ^a#^
5.0 mg/kg	190.61 ± 9.98 ^b^	150.22 ± 9.46 ^b#^	439.94 ± 23.52 ^b^	253.52 ± 13.85 ^a#^

Values are Mean ± SD. Values within the same column with different superscripts (^a^, ^b^) are significantly different at *p* < 0.05. ^#^ indicates significantly different from 3 weeks within the same exposure group at *p* < 0.05.

**Table 3:** DNA Methylation by Aflatoxin B1 Exposure Group at 3 Weeks of Age in Liver.

	*Tp53*					*H19*				
Exposure Group	Mean (SD)	N	ANOVA *p*-Value	Effect Estimate (SE)	*p*-Value	Mean (SD)	N	ANOVA *p*-Value	Effect Estimate (SE)	*p*-Value
Control	5.99 (1.46)	16	0.06	ref		49.56 (9.54)	18	0.0041	ref	
Low—0.5 mg/kg	4.82 (1.54)	16		−1.09 (0.34)	0.002	55.33 (4.60)	18		5.37 (2.56)	0.04
High—5.0 mg/kg	5.12 (1.19)	16		−0.91 (0.34)	0.01	56.80 (3.51)	17		6.88 (2.64)	0.0098

For each exposure group, Mean ± SD of percent methylation at each gene is shown along with a *p*-value from ANOVA comparing the groups. Mixed-effects regression was also performed, and effect estimates are shown for the exposure group. For *Tp53*, the outcome consists of repeat measures of DNA methylation at 9 CpG sites, and the model adjusts for offspring sex and CpG site. For *H19*, the outcome is repeat measures at 6 CpG sites, and the model adjusts for sex, site, and a random intercept for dam.

**Table 4:** DNA Methylation by Aflatoxin B1 Exposure Group at 3 Weeks and 3 Months of Age in Blood.

	*Tp53*					*H19*				
Exposure Group	Mean (SD)	N	ANOVA *p*-Value	Effect Estimate (SE)	*p*-Value	Mean (SD)	N	ANOVA *p*-Value	Effect Estimate (SE)	*p*-Value
3-weeks										
Control	3.10 (2.44)	18	0.07 *	ref		41.74 (2.68)	18	0.08	ref	
Low—0.5 mg/kg	4.55 (2.61)	18		1.69 (0.73)	0.02	40.79 (2.16)	18		−0.80 (1.04)	0.44
High—5.0 mg/kg	6.15 (4.86)	14		3.42 (0.78)	<0.0001	42.83 (2.88)	16		1.07 (1.08)	0.32
3-months										
Control	0.82 (1.20)	20	<0.001	ref		52.87 (16.08)	17	0.038	ref	
Low—0.5 mg/kg	2.48 (1.94)	23		1.52 (0.38)	0.0002	42.96 (6.40)	23		−10.7 (6.05)	0.08
High—5.0 mg/kg	4.53 (2.24)	13		3.11 (0.44)	<0.0001	41.18 (18.97)	15		−12.8 (6.59)	0.05

For each exposure group, Mean ± SD of percent methylation at each gene is shown along with a *p*-value from ANOVA comparing the groups. * Indicates Welch’s test was used instead of ANOVA because variances were unequal (Leven’s test *p* = 0.04). Mixed-effects regression was also performed, and effect estimates are shown for the exposure group. For *Tp53*, the outcome consists of repeat measures of DNA methylation at 9 CpG sites, and the model adjusts for offspring sex and CpG site. For *H19*, the outcome is repeat measures at 6 CpG sites, and the model adjusts for sex, site, and a random intercept for dam.

## Data Availability

The data presented in this study are available on request from the corresponding author.

## Supplementary Materials

The following are available online at https://www.mdpi.com/1660-4601/18/2/589/s1, Table S1: Primers for DNA Methylation Analysis via Pyrosequencing.

## References

[1] Gluckman P.D., Hanson M.A., Cooper C., Thornburg K.L. (2008). Effect of in utero and early-life conditions on adult health and disease. N. Engl. J. Med..

[2] Barker D.J., Osmond C., Forsen T.J., Kajantie E., Eriksson J.G. (2005). Trajectories of growth among children who have coronary events as adults. N. Engl. J. Med..

[3] Walker C.L., Ho S.-M. (2012). Developmental reprogramming of cancer susceptibility. Nat. Rev. Cancer.

[4] Sharma S., Kohli C., Johnson L., Bennet L., Brusselaers N., Nilsson P.M. (2020). Birth size and cancer prognosis: A systematic review and meta-analysis. J. Dev. Orig. Health Dis..

[5] Spector L.G., Puumala S.E., Carozza S.E., Chow E.J., Fox E.E., Horel S., Johnson K.J., McLaughlin C.C., Reynolds P., Von Behren J. (2009). Cancer risk among children with very low birth weights. Pediatrics.

[6] Ikeda H., Matsuyama S., Tanimura M. (1997). Association between hepatoblastoma and very low birth weight: A trend or a chance?. J. Pediatr..

[7] Zimmermann E., Berentzen T.L., Gamborg M., Sørensen T.I., Baker J.L. (2016). Sex-specific associations between birth weight and adult primary liver cancer in a large cohort of D anish children. Int. J. Cancer.

[8] Sandboge S., Perälä M.-M., Salonen M.K., Blomstedt P.A., Osmond C., Kajantie E., Barker D.J., Eriksson J.G. (2013). Early growth and non-alcoholic fatty liver disease in adulthood—the NAFLD liver fat score and equation applied on the Helsinki Birth Cohort Study. Ann. Med..

[9] Li E. (2002). Chromatin modification and epigenetic reprogramming in mammalian development. Nat. Rev. Genet..

[10] Reik W., Dean W., Walter J. (2001). Epigenetic reprogramming in mammalian development. Science.

[11] Silva Lima B., Van der Laan J.W. (2000). Mechanisms of Nongenotoxic Carcinogenesis and Assessment of the Human Hazard. Regul. Toxicol. Pharm..

[12] St-Pierre J., Hivert M.F., Perron P., Poirier P., Guay S.P., Brisson D., Bouchard L. (2012). IGF2 DNA methylation is a modulator of newborn’s fetal growth and development. Epigenet. Off. J. DNA Methylation Soc..

[13] Amarger V., Giudicelli F., Pagniez A., Parnet P. (2017). Perinatal high methyl donor alters gene expression in IGF system in male offspring without altering DNA methylation. Future Sci. OA.

[14] Agrogiannis G.D., Sifakis S., Patsouris E.S., Konstantinidou A.E. (2014). Insulin-like growth factors in embryonic and fetal growth and skeletal development (Review). Mol. Med. Rep..

[15] Jones P.A. (2012). Functions of DNA methylation: Islands, start sites, gene bodies and beyond. Nat. Rev. Genet..

[16] Dolinoy D.C., Huang D. (2007). Jirtle, R.L. Maternal nutrient supplementation counteracts bisphenol A-induced DNA hypomethylation in early development. Proc. Natl. Acad. Sci. USA.

[17] Montrose L., Padmanabhan V., Goodrich J.M., Domino S.E., Treadwell M.C., Meeker J.D., Watkins D.J., Dolinoy D.C. (2018). Maternal levels of endocrine disrupting chemicals in the first trimester of pregnancy are associated with infant cord blood DNA methylation. Epigenetics.

[18] Neier K., Cheatham D., Bedrosian L.D., Dolinoy D.C. (2019). Perinatal exposures to phthalates and phthalate mixtures result in sex-specific effects on body weight, organ weights and intracisternal A-particle (IAP) DNA methylation in weanling mice. J. Dev. Orig. Health Dis..

[19] Hernandez-Vargas H., Castelino J., Silver M.J., Dominguez-Salas P., Cros M.P., Durand G., Le Calvez-Kelm F., Prentice A.M., Wild C.P., Moore S.E. (2015). Exposure to aflatoxin B1 in utero is associated with DNA methylation in white blood cells of infants in The Gambia. Int. J. Epidemiol..

[20] Zhang Y.J., Rossner P., Chen Y., Agrawal M., Wang Q., Wang L., Ahsan H., Yu M.W., Lee P.H., Santella R.M. (2006). Aflatoxin B1 and polycyclic aromatic hydrocarbon adducts, p53 mutations and p16 methylation in liver tissue and plasma of hepatocellular carcinoma patients. Int. J. Cancer.

[21] Zhang Y.J., Wu H.C., Yazici H., Yu M.W., Lee P.H., Santella R.M. (2012). Global hypomethylation in hepatocellular carcinoma and its relationship to aflatoxin B(1) exposure. World J. Hepatol..

[22] Livingstone M.C., Johnson N.M., Roebuck B.D., Kensler T.W., Groopman J.D. (2017). Profound changes in miRNA expression during cancer initiation by aflatoxin B(1) and their abrogation by the chemopreventive triterpenoid CDDO-Im. Mol. Carcinog..

[23] Wu H.C., Wang Q., Yang H.I., Tsai W.Y., Chen C.J., Santella R.M. (2013). Global DNA methylation in a population with aflatoxin B1 exposure. Epigenetics.

[24] Kumi J., Mitchell N., Asare G., Dotse E., Kwaa F., Phillips T., Ankrah N. (2014). Aflatoxins and fumonisins contamination of home-made food (weanimix) from cereal-legume blends for children. Ghana Med. J..

[25] Benkerroum N. (2020). Aflatoxins: Producing-Molds, Structure, Health Issues and Incidence in Southeast Asian and Sub-Saharan African Countries. Int. J. Environ. Res. Public Health..

[26] Xue K.S., Tang L., Shen C.L., Pollock B.H., Guerra F., Phillips T.D., Wang J.-S. (2020). Increase in aflatoxin exposure in two populations residing in East and West Texas, United States. Int. J. Hyg. Environ. Health.

[27] Nazhand A., Durazzo A., Lucarini M., Souto E.B., Santini A. (2020). Characteristics, Occurrence, Detection and Detoxification of Aflatoxins in Foods and Feeds. Foods.

[28] Gruber-Dorninger C., Jenkins T., Schatzmayr G. (2019). Global mycotoxin occurrence in feed: A ten-year survey. Toxins.

[29] Zhang N.-Y., Qi M., Gao X., Zhao L., Liu J., Gu C.-Q., Song W.-J., Krumm C.S., Sun L.-H., Qi D.-S. (2016). Response of the hepatic transcriptome to aflatoxin B 1 in ducklings. Toxicon.

[30] Deng J., Zhao L., Zhang N.-Y., Karrow N.A., Krumm C.S., Qi D.-S., Sun L.-H. (2018). Aflatoxin B1 metabolism: Regulation by phase I and II metabolizing enzymes and chemoprotective agents. Mutat. Res. Rev. Mutat. Res..

[31] Benkerroum N. (2020). Chronic and acute toxicities of aflatoxins: Mechanisms of action. Int. J. Environ. Res. Public Health.

[32] Ferlay J., Soerjomataram I., Dikshit R., Eser S., Mathers C., Rebelo M., Parkin D.M., Forman D., Bray F. (2015). Cancer incidence and mortality worldwide: Sources, methods and major patterns in GLOBOCAN 2012. Int. J. Cancer.

[33] Rieswijk L., Claessen S.M., Bekers O., van Herwijnen M., Theunissen D.H., Jennen D.G., de Kok T.M., Kleinjans J.C., van Breda S.G. (2016). Aflatoxin B1 induces persistent epigenomic effects in primary human hepatocytes associated with hepatocellular carcinoma. Toxicology.

[34] Khlangwiset P., Shephard G.S., Wu F. (2011). Aflatoxins and growth impairment: A review. Crit. Rev. Toxicol..

[35] Fujitake S., Hibi K., Okochi O., Kodera Y., Ito K., Akiyama S., Nakao A. (2004). Aberrant methylation of SOCS-1 was observed in younger colorectal cancer patients. J. Gastroenterol..

[36] Steele R., Lane D. (2005). P53 in cancer: A paradigm for modern management of cancer. Surgeon.

[37] Rotimi O.A., Rotimi S.O., Oluwafemi F., Ademuyiwa O., Balogun E.A. (2016). Coexistence of aflatoxicosis with protein malnutrition worsens hepatic oxidative damage in rats. J. Biochem. Mol. Toxicol..

[38] Charni-Natan M., Aloni-Grinstein R., Osher E., Rotter V. (2019). Liver and Steroid Hormones—Can a Touch of p53 Make a Difference?. Front. Endocrinol..

[39] Goldstein I., Ezra O., Rivlin N., Molchadsky A., Madar S., Goldfinger N., Rotter V. (2012). p53, a novel regulator of lipid metabolism pathways. J. Hepatol..

[40] Lacroix M., Riscal R., Arena G., Linares L.K., Le Cam L. (2020). Metabolic functions of the tumor suppressor p53: Implications in normal physiology, metabolic disorders, and cancer. Mol. Metab..

[41] Berger C., Qian Y., Chen X. (2013). The p53-estrogen receptor loop in cancer. Curr. Mol. Med..

[42] Dinda S., Sanchez A., Moudgil V. (2002). Estrogen-like effects of thyroid hormone on the regulation of tumor suppressor proteins, p53 and retinoblastoma, in breast cancer cells. Oncogene.

[43] Rotimi O.A., Rotimi S.O., Duru C.U., Ebebeinwe O.J., Abiodun A.O., Oyeniyi B.O., Faduyile F.A. (2017). Acute aflatoxin B1–Induced hepatotoxicity alters gene expression and disrupts lipid and lipoprotein metabolism in rats. Toxicol. Rep..

[44] Rotimi O.A., Rotimi S.O., Goodrich J.M., Adelani I.B., Agbonihale E., Talabi G. (2019). Time-course effects of acute aflatoxin B1 exposure on hepatic mitochondrial lipids and oxidative stress in rats. Front. Pharmacol..

[45] Ugbaja R.N., Okedairo O.M., Oloyede A.R., Ugwor E.I., Akinloye D.I., Ojo O.P., Ademuyiwa O. (2020). Probiotics consortium synergistically ameliorates aflatoxin B1-induced disruptions in lipid metabolism of female albino rats. Toxicon.

[46] Supriya C., Girish B., Reddy P.S. (2014). Aflatoxin B1-induced reproductive toxicity in male rats: Possible mechanism of action. Int. J. Toxicol..

[47] Adedara I.A., Nanjappa M.K., Farombi E.O., Akingbemi B.T. (2014). Aflatoxin B1 disrupts the androgen biosynthetic pathway in rat Leydig cells. Food Chem. Toxicol..

[48] Chen X., Li C., Chen Y., Ni C., Chen X., Zhang L., Xu X., Chen M., Ma X., Zhan H. (2019). Aflatoxin B1 impairs leydig cells through inhibiting AMPK/mTOR-mediated autophagy flux pathway. Chemosphere.

[49] Cui H., Cruz-Correa M., Giardiello F.M., Hutcheon D.F., Kafonek D.R., Brandenburg S., Wu Y., He X., Powe N.R., Feinberg A.P. (2003). Loss of IGF2 imprinting: A potential marker of colorectal cancer risk. Science.

[50] Yoshimura H., Matsuda Y., Yamamoto M., Kamiya S., Ishiwata T. (2018). Expression and role of long non-coding RNA H19 in carcinogenesis. Front. Biosci..

[51] Raveh E., Matouk I.J., Gilon M., Hochberg A. (2015). The H19 Long non-coding RNA in cancer initiation, progression and metastasis—A proposed unifying theory. Mol. Cancer.

[52] Adetunji M., Atanda O., Ezekiel C.N., Sulyok M., Warth B., Beltrán E., Krska R., Obadina O., Bakare A., Chilaka C.A. (2014). Fungal and bacterial metabolites of stored maize (*Zea mays*, L.) from five agro-ecological zones of Nigeria. Mycotoxin Res..

[53] Grunau C., Clark S.J., Rosenthal A. (2001). Bisulfite genomic sequencing: Systematic investigation of critical experimental parameters. Nucleic Acids Res..

[54] Tost J., Gut I.G. (2007). Analysis of gene-specific DNA methylation patterns by pyrosequencing technology. Methods Mol. Biol..

[55] He X., Xie Z., Dong Q., Li J., Li W., Chen P. (2015). Effect of Folic Acid Supplementation on Renal Phenotype and Epigenotype in Early Weanling Intrauterine Growth Retarded Rats. Kidney Blood Press Res..

[56] Goodrich J.M., Sanchez B.N., Dolinoy D.C., Zhang Z., Hernandez-Avila M., Hu H., Peterson K.E., Tellez-Rojo M.M. (2015). Quality Control and Statistical Modeling for Environmental Epigenetics: A Study on in Utero Lead Exposure and DNA Methylation at Birth. Epigenetics.

[57] Kihara T., Matsuo T., Sakamoto M., Yasuda Y., Yamamoto Y., Tanimura T. (2000). Effects of prenatal aflatoxin B1 exposure on behaviors of rat offspring. Toxicol. Sci..

[58] Supriya C., Akhila B., Pratap Reddy K., Girish B., Sreenivasula Reddy P. (2016). Effects of maternal exposure to aflatoxin B1 during pregnancy on fertility output of dams and developmental, behavioral and reproductive consequences in female offspring using a rat model. Toxicol. Mech. Methods.

[59] Supriya C., Reddy P.S. (2015). Prenatal exposure to aflatoxin B1: Developmental, behavioral, and reproductive alterations in male rats. Sci. Nat..

[60] Turner P.C., Collinson A.C., Cheung Y.B., Gong Y., Hall A.J., Prentice A.M., Wild C.P. (2007). Aflatoxin exposure in utero causes growth faltering in Gambian infants. Int. J. Epidemiol..

[61] Lee B.J., Kim J.Y. (2020). Identification of metabolic syndrome using phenotypes consisting of triglyceride levels with anthropometric indices in Korean adults. BMC Endocr. Disord..

[62] Chatterjee C., Sparks D.L. (2011). Hepatic lipase, high density lipoproteins, and hypertriglyceridemia. Am. J. Pathol..

[63] Liu J., Zeng F.F., Liu Z.M., Zhang C.X., Ling W.H., Chen Y.M. (2013). Effects of blood triglycerides on cardiovascular and all-cause mortality: A systematic review and meta-analysis of 61 prospective studies. Lipids Health Dis..

[64] Abdel-Wahhab M.A., El-Nekeety A.A., Hathout A.S., Salman A.S., Abdel-Aziem S.H., Sabry B.A., Hassan N.S., Abdel-Aziz M.S., Aly S.E., Jaswir I. (2020). Bioactive compounds from Aspergillus niger extract enhance the antioxidant activity and prevent the genotoxicity in aflatoxin B1-treated rats. Toxicon.

[65] El-Nekeety A.A., Abdel-Azeim S.H., Hassan A.M., Hassan N.S., Aly S.E., Abdel-Wahhab M.A. (2014). Quercetin inhibits the cytotoxicity and oxidative stress in liver of rats fed aflatoxin-contaminated diet. Toxicol. Rep..

[66] Hu J., Zhang Z., Shen W.-J., Azhar S. (2010). Cellular cholesterol delivery, intracellular processing and utilization for biosynthesis of steroid hormones. Nutr. Metab..

[67] Giatti S., Diviccaro S., Serafini M.M., Caruso D., Garcia-Segura L.M., Viviani B., Melcangi R.C. (2020). Sex differences in steroid levels and steroidogenesis in the nervous system: Physiopathological role. Front. Neuroendocrinol..

[68] Trejter M., Hochol A., Tyczewska M., Ziolkowska A., Jopek K., Szyszka M., Malendowicz L.K., Rucinski M. (2015). Sex-related gene expression profiles in the adrenal cortex in the mature rat: Microarray analysis with emphasis on genes involved in steroidogenesis. Int. J. Mol. Med..

[69] Hasanzadeh S., Hosseini E., Rezazadeh L. (2011). Effects of Aflatoxin B1 on Profiles of Gonadotropic (FSH and LH), Steroid (Testosterone and 17β-Estradiol) and Prolactin Hormones in Adult Male Rat. Iran. J. Vet. Res..

[70] Abu El-Saad A.S., Mahmoud H.M. (2009). Phytic acid exposure alters aflatoxinB1-induced reproductive and oxidative toxicity in albino rats (*Rattus norvegicus*). Evid. Based Complement. Altern. Med..

[71] Amin Y., Mohamed R., Zakaria A., Wehrend A., Hussein H.A. (2019). Effects of aflatoxins on some reproductive hormones and composition of buffalo’s milk. Comp. Clin. Pathol..

[72] Onuzulu C.D., Rotimi O.A., Rotimi S.O. (2019). Epigenetic modifications associated with in utero exposure to endocrine disrupting chemicals BPA, DDT and Pb. Rev. Environ. Health.

[73] Flavahan W.A., Gaskell E., Bernstein B.E. (2017). Epigenetic plasticity and the hallmarks of cancer. Science.

[74] Darwiche N. (2020). Epigenetic mechanisms and the hallmarks of cancer: An intimate affair. Am. J. Cancer Res..

[75] Wang S., He Z., Li D., Zhang B., Li M., Li W., Zhu W., Xing X., Zeng X., Wang Q. (2017). Aberrant methylation of RUNX3 is present in Aflatoxin B(1)-induced transformation of the L02R cell line. Toxicology.

[76] Marrone A.K., Tryndyak V., Beland F.A., Pogribny I.P. (2016). MicroRNA Responses to the Genotoxic Carcinogens Aflatoxin B1 and Benzo[a]pyrene in Human HepaRG Cells. Toxicol. Sci..

[77] Tryndyak V., Kindrat I., Dreval K., Churchwell M.I., Beland F.A., Pogribny I.P. (2018). Effect of aflatoxin B(1), benzo[a]pyrene, and methapyrilene on transcriptomic and epigenetic alterations in human liver HepaRG cells. Food Chem. Toxicol..

[78] Zhang Y.J., Chen Y., Ahsan H., Lunn R.M., Chen S.Y., Lee P.H., Chen C.J., Santella R.M. (2005). Silencing of glutathione S-transferase P1 by promoter hypermethylation and its relationship to environmental chemical carcinogens in hepatocellular carcinoma. Cancer Lett..

[79] Zhang Y.J., Ahsan H., Chen Y., Lunn R.M., Wang L.Y., Chen S.Y., Lee P.H., Chen C.J., Santella R.M. (2002). High frequency of promoter hypermethylation of RASSF1A and p16 and its relationship to aflatoxin B1-DNA adduct levels in human hepatocellular carcinoma. Mol. Carcinog..

[80] Hussain S.P., Schwank J., Staib F., Wang X.W., Harris C.C. (2007). TP53 mutations and hepatocellular carcinoma: Insights into the etiology and pathogenesis of liver cancer. Oncogene.

[81] Menendez D., Inga A., Resnick M.A. (2006). The biological impact of the human master regulator p53 can be altered by mutations that change the spectrum and expression of its target genes. Mol. Cell. Biol..

[82] Hoyo C., Daltveit A.K., Iversen E., Benjamin-Neelon S.E., Fuemmeler B., Schildkraut J., Murtha A.P., Overcash F., Vidal A.C., Wang F. (2014). Erythrocyte folate concentrations, CpG methylation at genomically imprinted domains, and birth weight in a multiethnic newborn cohort. Epigenetics.

[83] LaRocca J., Binder A.M., McElrath T.F., Michels K.B. (2014). The impact of first trimester phthalate and phenol exposure on IGF2/H19 genomic imprinting and birth outcomes. Environ. Res..

[84] Lee H.S., Barraza-Villarreal A., Biessy C., Duarte-Salles T., Sly P.D., Ramakrishnan U., Rivera J., Herceg Z., Romieu I. (2014). Dietary supplementation with polyunsaturated fatty acid during pregnancy modulates DNA methylation at IGF2/H19 imprinted genes and growth of infants. Physiol. Genom..

[85] Soubry A., Murphy S., Huang Z., Murtha A., Schildkraut J., Jirtle R., Wang F., Kurtzberg J., Demark-Wahnefried W., Forman M. (2011). The effects of depression and use of antidepressive medicines during pregnancy on the methylation status of the IGF2 imprinted control regions in the offspring. Clin. Epigenet..

[86] Yang M.-L., Huang Z., Wang Q., Chen H.-H., Ma S.-N., Wu R., Cai W.-S. (2018). The association of polymorphisms in lncRNA-H19 with hepatocellular cancer risk and prognosis. Biosci. Rep..

