# Supplementary material for: Early Life Exposure to Aflatoxin B1 in Rats: Alterations in Lipids, Hormones, and DNA Methylation among the Offspring
Source: Int J Environ Res Public Health. 2021 Jan 12;18(2):589. doi: 10.3390/ijerph18020589 (PMC7828191; doi:10.3390/ijerph18020589)
Supplement: Supplementary file 1 [file ijerph-18-00589-s001.pdf]

Table S1. Primers for DNA Methylation Analysis via Pyrosequencing.

Gene	Genomic Location of Amplicon *	Forward Primer	Reverse Primer	Sequencing Primer	# CpG Sites	Details on region
Tp53	chr10: 56185594-56186023	TGGGGTTGGGATTAGGTTAGAAG	Biotin-ACTACACCTCCCAAAAACCT	GTAATTAAAAGTAAATAGAGGA	9	promoter
H19	chr1: 215749439-215749579	GTTTTTGGATTTTTAAATTAGTTAG	Biotin-TCCCCAAAAATTAACTACTTCTAAACT	GTTTTTGGATTTTTAAATTAGTTAG	6	imprinting control region (ICR) in the promoter

* From July 2014 Assembly (RGSC 6.0/rn6).
